# Nutrient reduction induced stringent responses promote bacterial quorum-sensing divergence for population fitness

**DOI:** 10.1038/srep34925

**Published:** 2016-10-07

**Authors:** Kelei Zhao, Xikun Zhou, Wujiao Li, Xiuyue Zhang, Bisong Yue

**Affiliations:** 1 Key Laboratory of Bio-resources and Eco-environment (Ministry of Education), College of Life Sciences, Sichuan University, Chengdu 610064, China; 2 State Key Laboratory of Biotherapy/Collaborative Innovation Center for Biotherapy, West China Hospital, Sichuan University, Chengdu 610064, China; 3 Sichuan Key Laboratory of Conservation Biology on Endangered Wildlife, College of Life Science, Sichuan University, Chengdu 610064, China

## Abstract

Bacteria use a cell-cell communication system termed quorum-sensing (QS) to adjust population size by coordinating the costly but beneficial cooperative behaviors. It has long been suggested that bacterial social conflict for expensive extracellular products may drive QS divergence and cause the “tragedy of the commons”. However, the underlying molecular mechanism of social divergence and its evolutionary consequences for the bacterial ecology still remain largely unknown. By using the model bacterium *Pseudomonas aeruginosa* PAO1, here we show that nutrient reduction can promote QS divergence for population fitness during evolution but requiring adequate cell density. Mechanically, decreased nutrient supplies can induce RpoS-directed stringent response and enhance the selection pressure on *lasR* gene, and *lasR* mutants are evolved in association with the DNA mismatch repair “switch-off”. The *lasR* mutants have higher relative fitness than QS-intact individuals due to their energy-saving characteristic under nutrient decreased condition. Furthermore an optimal incorporation of *lasR* mutants is capable of maximizing the fitness of entire population during *in vitro* culture and the colonization in mouse lung. Consequently, rather than worsen the population health, QS-coordinated social divergence is an elaborate evolutionary strategy that renders the entire bacterial population more fit in tough times.

Bacterial individuals communicate by using small, diffusible signal molecules in a process termed quorum-sensing (QS) for population fitness in a density-dependent manner^1^. The introduction of the concept QS has significantly enhanced the role of cooperative behaviors during bacterial social activities^2^,^3^. In a healthy cooperation system, the QS-intact individuals (cooperators) synthesize costly extracellular products (public goods) such as proteases, signal molecules and other chemical components with important biological activity for the fitness whole population^4^. However, such cooperation was considered unstable and vulnerable to invasion by the QS-deficient individuals (cheaters) who do not produce but still consume those public goods from others cooperating. Therefore, the current paradigm in sociomicrobiology is that increased frequency of QS-deficiency may compromise the fitness of entire population^3^,^4^,^5^,^6^.

*Pseudomonas aeruginosa* is a formidable Gram-negative opportunistic pathogen with large genome size normally causes acute or chronic infections in individuals who are burn patients, undergoing cancer chemotherapy, trauma, and especially those with genetic disease cystic fibrosis (CF)^7^. It is well-known that the QS system of *P. aeruginosa* is dominated by the central regulator LasR and triggered by the signal molecule, *N*-(3-oxododecanoyl)-HSL (3OC12-HSL)^8^,^9^,^10^. The *lasR* mutants with impaired QS function were defined as typical cheaters^11^. Clinical evidence showed that the *P. aeruginosa lasR* mutants were commonly identified from the lungs of CF patients who suffered a long-term pseudomonal infection^12^,^13^, indicating that social cheating can naturally happen in *P. aeruginosa* population during chronic lung infection.

Although several ingenious experiments have investigated the *de novo* emergence of *lasR* mutant in QS-required or rich medium^6^,^11^,^14^,^15^, the underlying molecular mechanism and its effect on population fitness still remain largely unknown. And these analyses may have neglected the role of nutrient supply in the development of social behaviors. Both bacterial proliferation and cooperation are energy cost, and the proportion of energy allocation into these behaviors may directly determine the fitness of population^16^. In a specific environment unit with less resource available, bacteria are likely to raise the investment into cooperation^17^. However, when the environmental nutrient level is insufficient to afford the intense QS with high cell density, the social conflict for public goods may promote the divergence of QS^18^. Previous mathematical modeling also suggested that food is more efficiently used when there is a famine^19^. Moreover, starvation is considered as the major signal guiding bacteria entry into stationary phase, and the altered global gene expression pattern and morphological change in this phase will increase bacterial viability against various environmental pressures^20^. Therefore, the emergence of QS-deficient individuals is probably approved by the population in response to nutrient reduction for further fitness.

To test our hypothesis, in this study we first perform a series of *in vitro* evolution of *P. aeruginosa* PAO1 in QS medium and rich medium with gradient nutrient levels. We find that nutrient reduction can promote bacterial social divergence but requiring adequate cell density. And this process is modulated by RpoS-dominated stringent response with the association of suppressed DNA mismatch repair (DMMR) system, as determined by the transcriptome analysis. We have also experimentally confirmed that *lasR* mutant has higher relative fitness than QS-proficient wild type (WT) PAO1 by escaping from the metabolic burden, and the incorporation of *lasR* mutants at optimal proportion can improve the fitness of entire population under nutrient decreased conditions and in the lungs of acutely and chronically infected mouse models.

## Results

### Nutrient reduction promotes bacterial social divergence during evolution

To verify the idea that nutrient reduction may serve as selection pressure and promote the *de novo* emergence of QS-deficient individuals, we performed a series of long-term evolution of WT PAO1 in QS-required and rich medium containing gradient concentrations of nutrient levels.

### *In vitro* evolution in QS medium

Most of the previous evolutionary screenings of *lasR* mutants were performed in QS medium, and they concluded that *lasR* mutants can emerge under culture conditions that require QS-dependent extracellular products for growth^6^,^11^,^15^. In this study, we further extended this conclusion by culturing WT PAO1 in M9 medium containing gradient concentrations of caseinate with or without pre-added 3OC12-HSL, to investigate the relationship between nutrient supply and QS divergence. In M9-caseinate medium (QS medium), caseinate as the sole carbon source should be broken down by the QS-dominated elastase (encoded by *lasB* gene) before use^1^. WT PAO1 can grow in this medium by developing QS, whereas *lasR* mutants can only grow by filching the elastase produced by WT PAO1. Previous studies demonstrated that exogenous QS signal could increase elastase production^1^, and this suggested that the pre-added QS signal may also contribute to QS divergence under nutrient reduction conditions.

**Figure 1:**
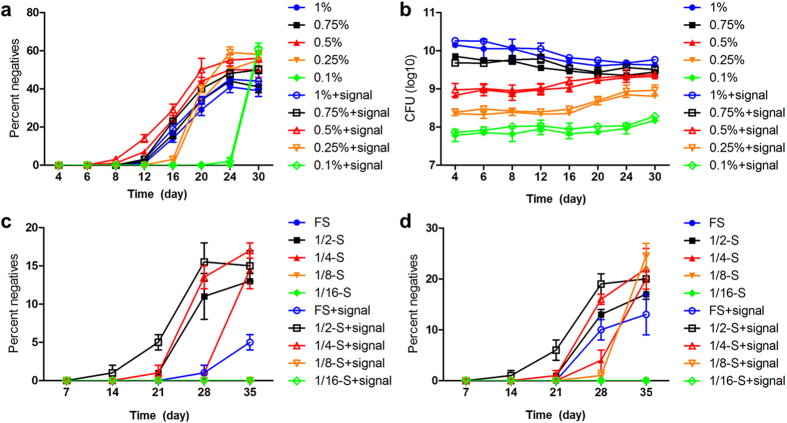
Nutrient reduction promotes *lasR* mutant emergence during *in vitro* evolution of WT PAO1. (**a**) Long-term evolution assays were performed in gradient QS medium with or without pre-added QS signal 3OC12-HSL to detect the proportions of the strains that failed to grow in M9-adenosine medium (Y-axis), and the cell density was measured before the starting of next cycle (**b**). The evolutionary screening was also performed in gradient MOPS-buffered LB broth. The experiments were designed as routinely shaking (**c**) and biofilm-related static cultures (**d**). FS, full-strength LB broth; 1/2-S, 1/2-strength LB broth; 1/4-S, 1/4-strength LB broth; 1/8-S, 1/8-strength LB broth; 1/16-S, 1/16-strength LB broth.

As shown in Fig. 1a, QS-deficient strains were first isolated from the culture with 0.5% caseinate on day 8, followed by from 0.75% and 1% caseinate medium on day 12. This result suggested that the time duration of QS divergence can be shortened with appropriately decreased nutrient supplies during evolution, as our screening and previous reports showed that *lasR* mutant would emerge after 10–15 days in 1% caseinate medium^6^,^11^,^21^. Moreover, the pre-addition of QS signal could elevate the proportions of QS-deficient strains among each culture. The *lasR* genes of a total of 50 adenosine growth-negative isolates were sequenced, finally, 3 kinds of point mutations and 1 truncation in transcription initiation region (*lasR1, lasR2, lasR3*, and *lasR4*) were identified and all the isolates had a LasR-null phenotype (Supplementary Table 1).

Surprisingly, QS-deficient strains of 0.25% and 0.1% caseinate medium with pre-added signal were identified on day 16 and 24, and that from the culture without pre-added signal were even later. We speculated that this might because the development of QS is cell density dependent (Fig. 1b). The low cell density caused by insufficient nutrient supplies would decrease QS activity^1^, and therefore restricted QS divergence. This is also accordant to the fact that there are scarce *lasR* mutants isolated from natural environments. The fractions of QS-deficient strains increased as culturing progressed and finally reached a stable equilibrium with the QS-intact counterparts, and the highest final proportion of QS-deficient strains was observed in 0.1% caseinate medium, followed by 0.25%, 0.5%, 0.75% and 1% culture (Fig. 1a). A recent study suggested that this equilibrium is dominated by a sort of “policing”, where the QS-intact individuals will use the second QS system, the Rhl system, to constrict QS-deficient individuals increasing by producing toxic cyanide^21^.

### *In vitro* evolution in rich medium

Although rich medium were considered as having equal selective advantage on QS-intact and -deficient individuals^6^,^14^, we were attracted by the result that *lasR* mutant reached a slightly lower maximal cell density than WT PAO1^14^, this indicated that a proficient QS system may also benefit population fitness in rich medium. We then repeated the *in vitro* evolution assay by culturing WT PAO1 in gradient dilutions of 3-(N-morpholino)-propanesulfonic acid (MOPS, pH 7.0)-buffered (to eliminate the effect of pH variation) LB broth (rich medium). This experiment was also repeated in a biofilm-related static culture, because biofilms are considered as efficient defense system that separating the bacterial population into units, and multiple lines of evidences had shown that *lasR* mutants are often isolated from biofilms in chronically infected patients with CF^22^,^23^.

When WT PAO1 was cultured in gradient dilutions of LB broth, although the growth rates and final cell densities were varied, the stationary phases were observed after 20 h (Supplementary Fig. 1). And we surprisingly noticed that the liquid color of 1/2-strength LB became visibly green compared with that of other dilutions after the 1 cycle (Supplementary Fig. 2), indicating a hyperactive QS system at this nutrient level and as confirmed by measuring the expression of *lasB* gene (Supplementary Fig. 3). These interesting findings suggested that the social cooperation in rich medium can be magnified by appropriately reducing the nutrient level. Accordingly, QS-deficient strains of shaking cultures were first identified from the 1/2-strength LB broth without pre-added QS signal on day 21, followed by 1/4-strength LB broth on day 28. No *lasR* mutant was identified in full-strength LB. When QS signal was pre-added, *lasR* mutants were also first identified from the 1/2-strength LB broth on day 14, followed by 1/4- and full-strength LB broth on day 21 and 28, respectively (Fig. 1c). For the biofilm-related selection, similar time points of *lasR* mutant emergences as in shaking culture were observed (Fig. 1d). After sequencing of *lasR* genes in 50 putative *lasR* mutants, 3 kinds of point mutation and 1 truncation in transcription initiation region (*lasR5, lasR6, lasR7*, and *lasR8*) were identified and all the isolates had a LasR-null phenotype (Supplementary Tables 2 and 3). Thus, these findings confirmed that the evolvement of bacterial social divergence can be promoted under nutrient reduced conditions but requiring adequate cell density to guarantee the successful development of QS.

### RpoS-dominated stringent response and DMMR “switch-off” contribute to social divergence

**Figure 2:**
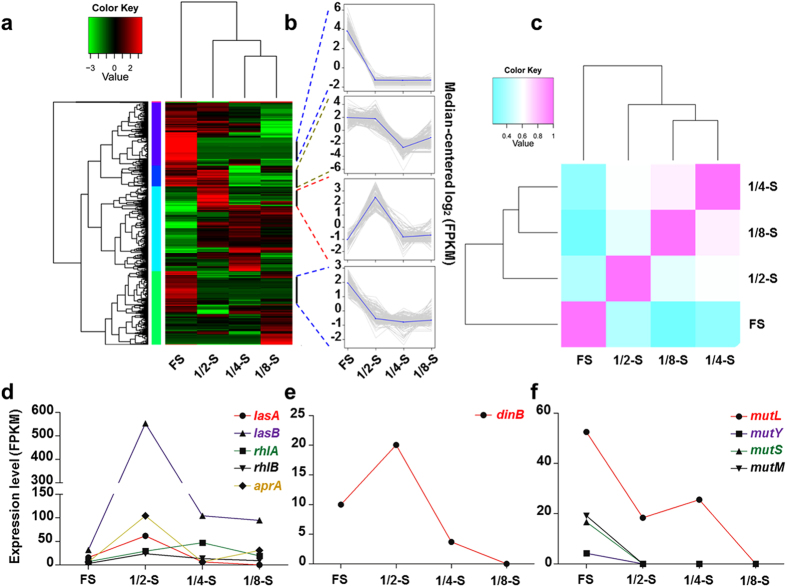
Global transcriptional differences of WT PAO1 cultured in gradient dilutions of LB broth. (**a**) Hierarchical clustering showing the relative expression levels of each transcript (rows) in each sample (column). (**b**) Transcript clusters extracted from the hierarchical clustering with R. X axis: samples; Y axis: median-centered log_2_ (FPKM). Gray lines, individual transcripts; blue lines, average expression values per cluster. (**c**) Heat map showing the hierarchically clustered Spearman correlation matrix that resulted from comparison of the transcript expression values for each pair of samples. Genes were considered overexpressed if the *P* value for their differential abundance between two samples was <0.05. (**d–f**) Expression levels of typical genes based on the values of FPKM.

To reveal the underlying molecular mechanism of bacterial social divergence, we then analyzed the global expression profiles of WT PAO1 under different nutrient levels. We also chose gradient dilutions of LB broth, because the expression of QS-related genes might be significantly changed in QS medium^11^. Total RNA of each culture was isolated at stationary phase and then conducted for RNA-Sequencing followed by differential expression analysis (Fig. 2a–c). Notably, cells grown in 1/2-strength LB broth showed elevated expression levels of QS-inducible genes and several exoproducts (Fig. 2d and Supplementary Table 4). This was also accordant to the visibly green color of 1/2-strength LB culture and hyperactive QS system (Supplementary Figs 2 and 3). In particular, WT PAO1 in the 1/2-strength LB medium showed significantly up-regulated expression of DinB (DNA polymerase IV) and down-regulated expression of DMMR-related genes (*mutL, mutY, mutS* and *mutM*) (Fig. 2e,f). DinB functions as powerful mutator contributing to mutagenesis, and in *Escherichia coli*, DinB expression is positively controlled by the stringent response regulator RpoS^24^. In contrast, MutS acts in replication-coupled mismatch repair can limit spontaneous mutations^25^.

**Figure 3:**
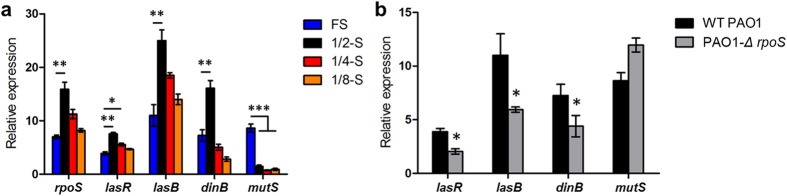
Gene expressions as determined by qPCR. (**a**) Gene expression of WT PAO1 cultured in gradient dilutions of LB broth. (**b**) Gene expression of WT PAO1 and *rpoS* mutant strain cultured in normal LB broth. Data are mean values ± SEM and are representative of three experiments. *P < 0.05, **P < 0.01, ***P < 0.001, one-way ANOVA (Tukey-Kramer post hoc analysis), or *t*-test.

**Figure 4:**
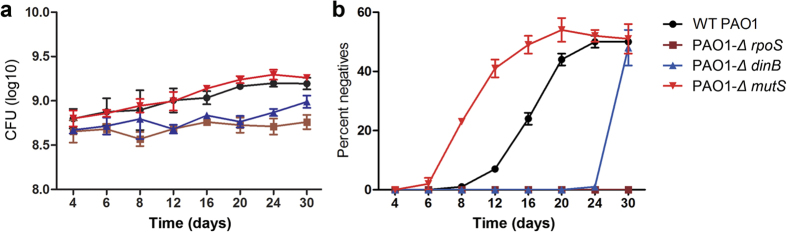
*In vitro* evolutionary screening of QS negative strains. WT PAO1, *rpoS* mutant, *dinB* mutant and *mutS* mutant strains were cultured in M9 medium supplied with 0.5% caseinate. Cultures were sub-cultured into fresh medium every 24 h. Y-axis indicates the proportions of the strains that failed to grow in M9-adenosine medium. Data are mean values ± SEM and are representative of three experiments.

To prevent the inevitable bias of transcriptome screening, the results were validated in more detail using quantitative PCR. The expression of RpoS in 1/2-strength LB broth was approximately two-fold greater than in full-strength culture. DinB expression was significantly increased in 1/2-strength culture (*P* < 0.01), and the expression of MutS was decreased (*P* < 0.001) (Fig. 3a). When the *rpoS* gene was knocked out, the expression levels of *lasR, lasB* and *dinB* were significantly decreased (*P* < 0.05) (Fig. 3b), and these results were also accordant to the previous findings that LasR is positively regulated by RpoS^26^. RpoS triggered *lasR* expression may contribute to explore potential nutrient factors by producing several exoproteases^20^. To confirm the roles of RpoS and DMMR system in the evolvement of QS divergence, we then performed a long-term culture of PAO1-*Δ rpoS*, PAO1-*Δ dinB*, and PAO1-*Δ mutS* in M9 medium containing 0.5% caseinate. As expected, comparing with WT PAO1, the *lasR* gene was more error-prone in *mutS* mutant but more conserved in *dinB* mutant and *rpoS* mutant strains (Fig. 4). Therefore, our results here confirmed that nutrient reduction induced RpoS-dominated stringent response and DMMR “switch-off” jointly promote the mutagenesis of *lasR* gene.

### LasR mutants consume less energy but more adapt to nutrient decreased condition

We then attempted to investigate why the *lasR* mutants can be frequently isolated from stressed *P. aeruginosa* population. The emergence of *lasR* mutants during evolution suggested that an intense QS system may not always be favored, especially when the population is undergoing nutrient reduction and insufficient to support continually increased social cooperation. The *lasR* mutant with impaired QS system may have higher relative fitness than QS-intact WT individuals by escaping from metabolic burden but benefit from others cooperating under the conditions with decreasing nutrient supplies.

**Figure 5:**
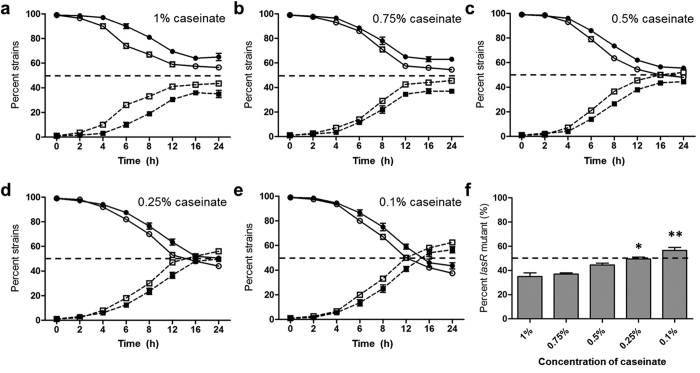
Nutrient reduction is beneficial for the fitness of *lasR* mutants. (**a**–**e**) Social divergence could be enhanced with decreasing nutrient supplies. Mixture of WT PAO1 and *lasR* mutant (99:1) were co-cultured in gradient M9-caseinate medium with (open symbols) or without (filled symbols) pre-added QS signal. Y-axis indicates the proportions of WT PAO1 and *lasR* mutant at designated time points. (**f**) Final proportions of *lasR* mutants among each nutrient level (without pre-added QS signal) after 36 h. Dash-line indicates the value of 50%. Mean values ± SEM of one experiment are shown and are representative of three independent experiments. *P < 0.05, **P < 0.01, one-way ANOVA (Tukey-Kramer post hoc analysis).

We tested this hypothesis by co-culturing WT PAO1 and the *lasR* mutant counterpart (99:1) in gradient QS medium. As shown in Fig. 5a–e, the proportions of WT PAO1 and *lasR* mutant showed a convergent trend with time, and this trend could be improved by pre-addition of QS signal and decreasing caseinate supplies. The final proportion of *lasR* mutant became higher than WT PAO1 (*P* < 0.05) when the concentrations of caseinate were <0.25% (wt/v) (Fig. 5f). The relative fitness of *lasR* mutants was increased along with decreasing nutrient levels and the value (*v*) became >1.0 when caseinate levels were <0.25% (Supplementary Fig. 4). This indicated that the relative growth rate of *lasR* mutants is lower than WT PAO1 in high nutrient levels but faster when resource is decreasing. Subsequently, the total ATP productions of WT PAO1 and *lasR* mutant in the culture with gradient nutrient levels were measured to investigate the underlying mechanism of this growth divergence. Because *lasR* mutant cannot grow in QS medium without QS-intact counterpart, casamino acids (CAA, the product of caseinate digested by elastase) were used instead of caseinate as the sole carbon source. Theoretically, if WT PAO1 showed higher ATP production level than *lasR* mutant in M9-CAA medium, the ATP production of WT PAO1 individual should be much higher than in *lasR* mutant when they were co-cultured in M9-caseinate medium. The final population sizes of WT PAO1 in 0.25% and 0.1% CAA medium were slightly lower than *lasR* mutant (Supplementary Fig. 5a), and when the cell densities were equalized, the ATP production of individual WT PAO1 in any CAA concentration was significantly greater than that in *lasR* mutant (*P* < 0.01) (Supplementary Fig. 5b). Therefore, these results provided detailed evidence that the emergence of *lasR* mutants is favored by the population to improve entire fitness using an energy-saving strategy to ease the cooperative burden under nutrient reduction.

### Incorporation of QS-deficient individuals at optimal proportion maximizes population fitness

**Figure 6:**
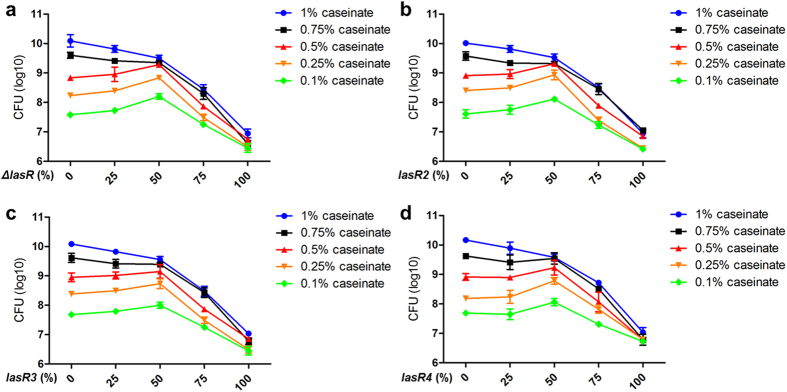
Incorporation of *lasR* mutant at appropriate proportion can maximize population size under nutrient decreased conditions. Different mixtures of WT PAO1 with *lasR* deletion mutant (**a**), as well as with the randomly selected *lasR* mutants *lasR2* (**b**), *lasR3* (**c**), and *lasR4* (**d**) in this study, were inoculated into gradient M9-caseinate medium and co-cultured for 36 h, respectively. Mean values ± SEM of one experiment are shown and are representative of three independent experiments. The experiments of panels **a**, **b** and **d** showed significantly (*P* < 0.05) increased cell density when caseinate levels were lower than 0.5% and the frequency of cheater (*lasR* mutant) is 50%.

By co-culturing different mixtures of WT PAO1 with *lasR* mutants, as well as with the randomly selected *lasR* mutants (*lasR2, lasR3,* and *lasR4*) in this study, we found that in the culture with high caseinate level, the whole population size would be shrunken or even collapsed with increasing proportions of *lasR* mutants (Fig. 6). Notably, when the co-culture was tested with decreasing nutrient levels and the proportion of *lasR* mutants was about 50%, the whole population size could be maximized. These results suggested that the incorporation of *lasR* mutants at appropriate proportion can maximize population fitness under nutrient decreased condition.

**Figure 7:**
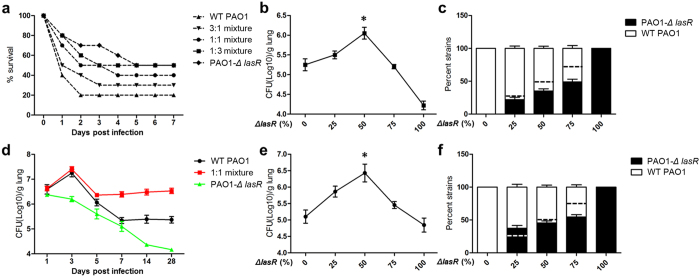
Optimal mixture of *lasR* mutants and WT PAO1 maximizes population fitness in mouse lung. (**a**) Survival rate of mice after acutely infected with gradient mixtures of WT PAO1 and *lasR* mutant. The residual CFUs (**b**) and final proportions of WT PAO1 and *lasR* mutant (**c**) in acutely infected mouse lungs were enumerated by combined using of LB and M9-adenosin agar on day 5. (**d**) Residual CFUs in the lungs of chronically infected mouse models. The total residual CFUs (**e**) and proportions of WT PAO1 and *lasR* mutant (**f**) in chronically infected mouse lungs were measured on day 14. Black or white dashed lines indicate the initial proportions of *lasR* mutants. Mean values ± SEM of one experiment are shown and are representative of three independent experiments. *P < 0.05, One-way ANOVA (Tukey-Kramer post hoc analysis).

We then set out to investigate the possibility that an optimal proportion of QS-intact and -deficient individuals might be beneficial for population fitness during infection. We developed acutely and chronically infected mouse models by intranasally instilling different mixtures of WT PAO1 and *lasR* mutants. For the acute infection model, the survival rate of mice became stable post 5 day’s infection in any case, and the incorporation of *lasR* mutants caused decreased lethality compared with pure WT PAO1 (Fig. 7a). The residual population that escaped from host lung clearance was maximized when the infection was started with 1:1 mixture of WT PAO1 and *lasR* mutants (*P* < 0.05) (Fig. 7b). Compared with the initial instilled proportions, the final proportions of *lasR* mutants were decreased in all the cases that mice were instilled with different mixtures (Fig. 7c), and this suggested that *lasR* mutants have a lower fitness than WT PAO1 during acute infection in mouse lungs.

For the chronic infection model, the lung residual CFUs became relatively stable after 7 days except the group infected with pure *lasR* mutant (Fig. 7d). Such stability indicates the establishment of chronic infection^27^. The total population size and composition of *P. aeruginosa* in mouse lung were measured on day 14. Accordingly, the maximal population size was observed in the infection started with 1:1 mixture of WT PAO1 and *lasR* mutants (*P* < 0.05) (Fig. 7e). Compared with the initial instilled proportions, the final proportion of *lasR* mutants was increased in the case that instilled with the mixture containing 25% *lasR* mutant. This suggested that *lasR* mutants have a higher fitness than WT PAO1 during chronic infection in mouse lungs. The final proportions of WT PAO1 and *lasR* mutants were around 50% in the cases that mice were instilled with different mixtures (Fig. 7f). This result combined with the final proportions of *lasR* mutants in long-term *in vitro* evolution, indicating that the optimal ratio of QS-intact/-deficient individuals for maximizing population fitness might be about 1:1. Consequently, we concluded that the incorporation of *lasR* mutants into *P. aeruginosa* population units at optimal proportion can maximize population fitness in the colonization of mouse lung, especially during chronic infection.

## Discussion

Although the living environments of bacteria are extensive and complex, the flexible genetic characteristic of bacteria may remedy their inferior in cell structure under various environmental pressures. The discovery of QS-coordinated cooperative and competitive behaviors makes the nutrient acquisition and allocation of bacteria more mysterious and attractive^28^. In this study, we provide experimental evidences regarding the potential molecular mechanism and significance of bacterial QS divergence during evolution.

**Figure 8:**
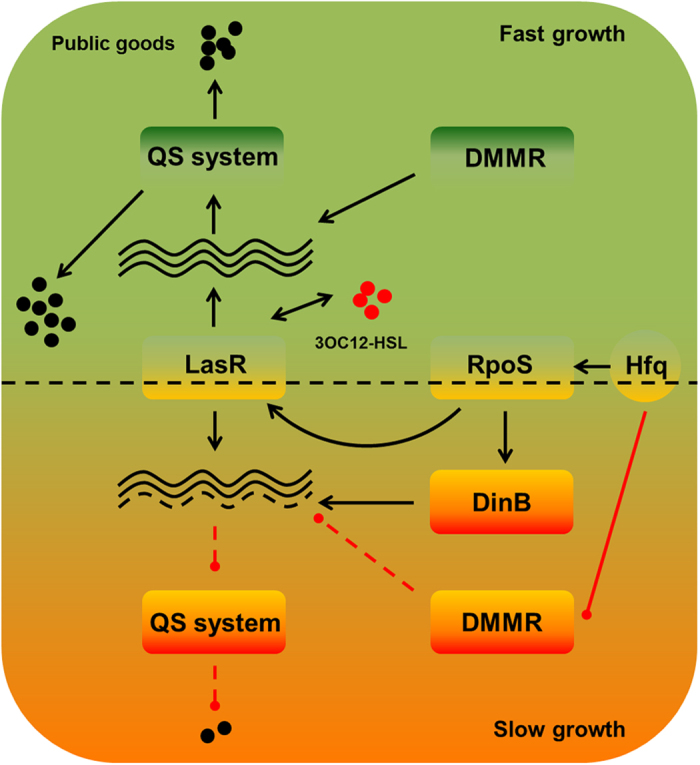
Schematic of nutrient reduction promoted mutagenesis of *lasR* gene of *P. aeruginosa*. Briefly, an integrated *las* QS system under DMMR system supervision can guarantee the intense social cooperation for fast population growth under nutrient abundant condition. Along with nutrient decreasing, the RpoS-dominated stringent response can promote *lasR* expression to explore potential available nutrient factors. Meanwhile, the increased expression of mutator DinB and suppressed DMMR system can jointly enhance bacterial genomic plasticity for further adaption. Mutagenesis in *lasR* gene can solve the dilemma that insufficient environmental resources can be explored for further costly cooperation and the imposed pressure to cooperate from RpoS on LasR.

On the basis of current molecular and growth evidences, we conclude that nutrient reduction can promote *de novo* emergence of *P. aeruginosa lasR* mutants under joint effect of RpoS-dominated stringent response and DMMR “switch-off” (Fig. 8). Since LasR plays a central role in the QS cascade, a healthy *las* QS system under DMMR system supervision can guarantee the intense social cooperation for population fitness under nutrient abundant condition^1^. However, when the decreased nutrient level cannot support the intense cooperation of the population with high cell density, RpoS will be activated and guiding the entry of population into stationary phase^20^. The RpoS-dominated stringent response can promote *lasR* expression to explore potential available nutrient factors by producing more exoproteases (Figs 2d and 3). Therefore, the QS-intact individuals will be caught in a dilemma against continuously decreased nutrient level: insufficient environmental resources can be explored for further highly developed costly cooperation and the imposed pressure to cooperate from RpoS on LasR. On the other hand, increased expression of mutator DinB under RpoS regulation and Hfq-suppressed DMMR system can jointly enhance bacterial genomic plasticity preparing for further adaption in stressed environments^29^ (Figs 2 and 4). Mutagenesis of *lasR* gene which is suffering high selection pressure during evolution can perfectly solve this dilemma, as we have noticed that the stable final population size during evolution was expanded with increasing proportions of QS-deficient individuals under nutrient reduced conditions (Fig. 1b).

We did not test the possibility that nutrient reduction may also cause mutagenesis of other genes during evolution but only in *lasR*, because this study mainly discussed the relationship between nutrient supply and QS system. Previous study using genomic sequencing showed that *lasR* mutant *P. aeruginosa* frequently exists in the lungs of CF patients, as well as mutations in other genes with various functions such as antibiotic resistance and virulence^13^. Additionally, *P. aeruginosa* from 36% of the CF patients (n = 30) who suffered from pseudomonal infection for years (especially more than 10 years) were hypermutable, whereas this hypermutable feature was not observed in the *P. aeruginosa* isolated from the lungs of acutely infected non-CF patients (n = 75)^12^,^13^. Subsequent studies showed that the frequency of *lasR* variants was increased in a *mutS* mutant strain with hypermutable features^30^,^31^. These findings also confirmed the association between suppressed function of DMMR system and mutagenesis of *lasR* gene. The *lasR* mutants of this study and those identified by Sandoz *et al*.^11^ were generated from a single WT colony and did not appear to be hypermutable strains as their mutation frequencies were lower than the criterion for hypermutability (Supplementary Table 5). Hypermutable strains will probably emerge after relatively longer culture periods under conditions of stringent response and DMMR “switch-off”.

Comparing with WT PAO1, the productivity of extracellular proteins of *lasR* mutant is decreased, and this can increase population fitness under limited resources during evolution by reducing the synthetic burden on the cell^32^. In the conditions with abundant resource supplies, increasing proportions of cheaters will decrease the whole population productivity^33^. On the contrary, as previously predicted in yeast, food resource will be more efficiently used and optimal mixture of cheaters and cooperators may maximize population benefits^19^. In this study, we found that the population fitness of *P. aeruginosa* is dominated by the levels of nutrient supplies and the proportions of *lasR* mutant cheaters in QS medium (Figs 1b and 6). Specifically, the emergence of cheaters with energy-saving characteristic during evolution is favored by the population to adapt the stationary phase with less resource available. The *lasR* mutants also possess several superior characteristics such as the utilization of particular carbon and nitrogen sources, increased antibiotic resistant ability, as well as avoid cell lysis^14^,^34^. All of these evolved phenotypes significantly contribute to the adaption of *P. aeruginosa* in environment of host lung. Because the environment of lung is more complex than *in vitro*, bacterial population may undergo various stresses such as high oxygen content and host immune system^35^. It is hard to provide a convinced mechanism for the finding that optimal mixture of QS-intact and -deficient individuals will benefit the fitness of whole population *in vivo*, but at least, the energy-saving characteristic of *lasR* mutants may play an important role. Further more comprehensive analyses on the basis of evolved superior characteristics of *lasR* mutants and cell-cell interactions as well as the various host tissue pressures may help to reveal the intrinsic mechanism.

In conclusion, the QS divergence of bacteria is an elaborate evolutionary strategy which can significantly increase the fitness of entire population in tough times. Our current findings enrich the concept of sociomicrobiology and open a new avenue for further investigation of QS-coordinated social divergence, which may also have implications in the development of novel antivirulence strategies for disrupting cell-cell communication in chronic lung infections.

## Materials

### Bacterial strains

Wild type *P. aeruginosa* PAO1, QS-deficient *P. aeruginosa* strain PAO1-*ΔlasR*, and the *rpoS* mutant strain PAO1-*ΔrpoS* were gifted from Dr. M. Schuster (Oregon State University, Corvallis, OR, USA) and Dr. S. Lory (Harvard Medical School, Boston, MA, USA)^26^. PAO1-*ΔdinB* and PAO1-*ΔmutS* were gifted from Dr. D. Wozniak (Ohio State University, Columbus, OH, USA)^24^. LB broth or designated medium were inoculated with the various strains and incubated at 37 °C.

### *In vitro* evolution

Long-term evolution of WT PAO1 was conducted to select *lasR* mutants. Equal amount (1.0 × 10^7^ CFU/ml) of WT PAO1 (which was generated from a single WT colony) was cultured in M9 medium containing different concentrations of caseinate (1%, 0.75%, 0.5%, 0.25%, and 0.1%), and in MOPS-buffered LB broth with gradient dilutions (full-, 1/2-, 1/4-, 1/8-, and 1/16-fold) at 37 °C. All the experiments were performed with shaking (220 rpm) and with or without pre-added QS signal (20 μM 3OC12-HSL). Medium of each culture was refreshed at 24 h interval. For biofilm-related selection, WT PAO1 was inoculated to sterile 12-well polystyrene plates with LB broth (strengths: full, 1/2, 1/4, 1/8, and 1/16) with or without pre-added QS signal. Glass cover slips (20 mm × 20 mm) were used to collect biofilms, and the plates were incubated at 37 °C without shaking. Culture liquids were removed at 24 h interval, and the same size (10 mm × 10 mm) of biofilm was cut from the same portion of glass cover slips for further identification. All the experiments were repeated for 3 times.

To investigate the roles of stringent response and DMMR system in *lasR* mutation, the *rpoS* mutant, *dinB* mutant and *mutS* mutant strains of WT PAO1 were also conducted for long-term evolution in M9 medium supplied with 0.5% caseinate. Bacteria from M9-caseinate cultures were harvested on days 4, 6, 8, 12, 16, 20, 24, and 30 for further screening of *lasR* variants. Bacteria from LB broth were harvested on days 7, 14, 21, 28 and 35. The selection and identification of *lasR* mutants were performed as previously described^11^. This experiment was also repeated for 3 times independently.

### Mutation frequencies of *lasR* mutants

To investigate whether the identified *lasR* mutants were hypermutable, their mutation frequencies were determined by exposing to rifampin as previously described by Ciofu *et al*.^36^. An isolate was considered hypermutable if the mutation frequency was 20 times higher than that of WT PAO1. All the experiments were repeated for 3 times.

### Transcriptome analysis

For global transcriptional analysis, WT PAO1 were cultured in MOPS-buffered LB broth of various strengths (full-, 1/2-, 1/4-, and 1/8-strength). Total RNA was isolated by TRIzol^®^ (Invitrogen) reagents at stationary phase of each culture after 24 h, and the RNA samples from 3 independent cultures were mixed to minimize the deviation of RNA-Seq. The cDNA libraries were constructed and then sequenced using Illumina Hiseq2000 technology. We employed mRNA-Seq for differential expression analysis based on the genome sequence and gene profiles of *P. aeruginosa* PAO1 (GenBank accession number: AE004091). The software Tophat^37^ was used to map mRNA sequences to the genome, and subsequently the combination of SOAP2 program^38^ and Cufflinks^39^ was used to calculate the expected fragments per kilobase of transcript per million fragments (FPKM) sequenced. Finally, the differentially expressed transcripts were presented and analyzed by edgeR^40^. The gene expression profiles of *P. aeruginosa* PAO1 have been deposited at DDBJ/EMBL/GenBank under the accessions PRJNA264943.

### Quantitative PCR

Total RNA was extracted as described above, and quantitative PCR was performed using the QIAGEN OneStep RT-PCR Kit per the manufacturer’s instructions. The designs of specific primers used in this study (Supplementary Table 6) were based on the consensus of sequences that are deposited in GenBank. Gene expression was calculated by the 2^−*ΔCT*^ or 2^−*ΔΔCT*^ method^41^ using 16S rRNA as reference.

### Competition experiments

LasR mutant and its parent WT PAO1 were co-cultured in 4 ml M9 medium^11^ containing gradient concentrations of caseinate (1%, 0.75%, 0.5%, 0.25%, and 0.1%) with or without the addition of 20 μM 3OC12-HSL. The cells of overnight cultured WT PAO1 and *lasR* mutant, which were started from the inoculation of single colony, were harvested and diluted to optical density (OD600) of 0.01 (corresponding to 2.0 × 10^7^ CFU/ml). The initial *lasR* mutant to WT PAO1 ratio was 1:99 (1.0 × 10^7^ CFU/ml) and then co-cultured for 36 h. Subsequently, 1 ml of culture liquid was used for plating on LB agar after appropriate dilution. A total of 100 colonies were randomly picked and plated on M9 adenosine agar to enumerate the CFUs of WT PAO1, because *lasR* mutants are failed to grow in this medium^14^. Subsequently, a 1:1 mixture of *lasR* mutant and WT PAO1 was inoculated into gradient M9-caseinate medium and cultured for 36 h to determine the relative fitness of cheaters. The relative fitness (*v*) was calculated by comparing the initial and final frequencies by using the equation *v* = [x_1_ (1 − x_0_)/x_0_ (1 − x_1_)], where x_0_ is the initial proportion in the population and x_1_ is their final proportion. The value of relative fitness could indicates the growth rate, for example, *v* = 2 means that the growth rate of *lasR* mutant was twice as fast as WT PAO1^3^,^15^. To investigate the effect of *lasR* mutation on population fitness, gradient mixtures of WT PAO1 and *lasR* mutants (0, 25%, 50%, 75% and 100%) were inoculated into gradient M9-caseinate medium and cultured for 36 h. All the experiments were repeated for 3 times.

### Bioluminescence assay

To test the idea that *lasR* mutants who escape from QS burden will consume less energy in compare with WT PAO1 under nutrient reduced conditions, equal amount of WT PAO1 and *lasR* mutants were cultured in M9 medium supplied with different concentrations of CAA as the sole carbon source for 24 h. Production of total ATP during culture was measured by bioluminescence with the ATP Determination Kit (Molecular Probes), in accordance with the manufacturer’s instructions.

### Mouse models. Acute lung infection in mice

Gradient ratio of WT PAO1 and PAO1-*ΔlasR* (pure WT PAO1, 3:1, 1:1, 1:3, and pure *lasR* mutant) with equal total amounts of CFU 1.0 × 10^7^ were intranasally instilled into C57BL/6J female mice (Indianapolis, IN; weight, 20–25 g, 15 mice per group) that anesthetized by intraperitoneal injection of ketamine (50 mg/ml) and xylazine (5 mg/ml) in 0.9% NaCl. The survival rate was recorded for 7 days. In another independent experiment, mice were killed post 5 days infection and 0.1–0.2 g lung samples from the same part of mouse lungs were aseptically excised and homogenized in 0.9% NaCl. The homogenate was then appropriately diluted and plated onto LB and M9-adenosin agar medium for measurement of the total residual CFU and proportions of WT PAO1 and *lasR* mutant.

### Chronic lung infection in mice

Agar beads containing gradient ratio of WT PAO1 and PAO1-*ΔlasR* (pure WT PAO1, 3:1, 1:1, 1:3, and pure *lasR* mutant) were prepared and enumerated as previously described^27^. The agar beads were diluted into the same inoculum of 1.0–2.0 × 10^6^ CFU in 50 μl and then intranasally instilled into anesthetized C57BL/6J female mice (15 mice per group). Mice were killed after 1, 3, 5, 7, 14, and 28 days. The residual CFUs in mouse lungs and proportions of WT PAO1 and *lasR* mutant were measured as described in acute infection.

### Statistical analyses

Graphpad Prism version 5.0 (San Diego, CA) was used to conduct data analysis and statistical tests. Mean values were compared by using *t*-test or one-way ANOVA and a subsequent Tukey-Kramer post hoc test using a 95% confidence interval.

### Ethics statement

All experiments were performed in accordance with the relevant guidelines and regulations, and the entire animal experimental procedures were approved by the guidance of Institutional Animal Care and Use Committee of Sichuan University (Chengdu, China).

## Additional Information

**How to cite this article**: Zhao, K. *et al*. Nutrient reduction induced stringent responses promote bacterial quorum-sensing divergence for population fitness. *Sci. Rep.* **6**, 34925; doi: 10.1038/srep34925 (2016).

## Supplementary Material

Supplementary Information
