# Supplementary material for: Nutrient reduction induced stringent responses promote bacterial quorum-sensing divergence for population fitness
Source: Sci Rep. 2016 Oct 7;6:34925. doi: 10.1038/srep34925 (PMC5054682; doi:10.1038/srep34925)
Supplement: Supplementary Information [file srep34925-s1.doc]

**Article Title:** Nutrient reduction induced stringent responses promote bacterial quorum-sensing divergence for population fitness

**Authors:** Kelei Zhao^1,2^, Xikun Zhou^2^, Wujiao Li^1^, Bisong Yue^3^* & Xiuyue Zhang^1,3^*

**Affiliations**

^1^ Key Laboratory of Bio-resources and Eco-environment (Ministry of Education), College of Life Sciences, ^2^ State Key Laboratory of Biotherapy/Collaborative Innovation Center for Biotherapy, West China Hospital, ^3^ Sichuan Key Laboratory of Conservation Biology on Endangered Wildlife, College of Life Science, Sichuan University, Chengdu 610064, China.

* Correspondence and requests for materials should be addressed to: Xiuyue Zhang and Bisong Yue, E-mail: zhangxy317@126.com and bsyue@scu.edu.cn. Tel.: +86 28 85412057; Fax: +86 28 85414886. Address: No.24 South Section 1, Yihuan Road, Chengdu 610064, China.

**Supplementary Data**

**Supplementary Figures**


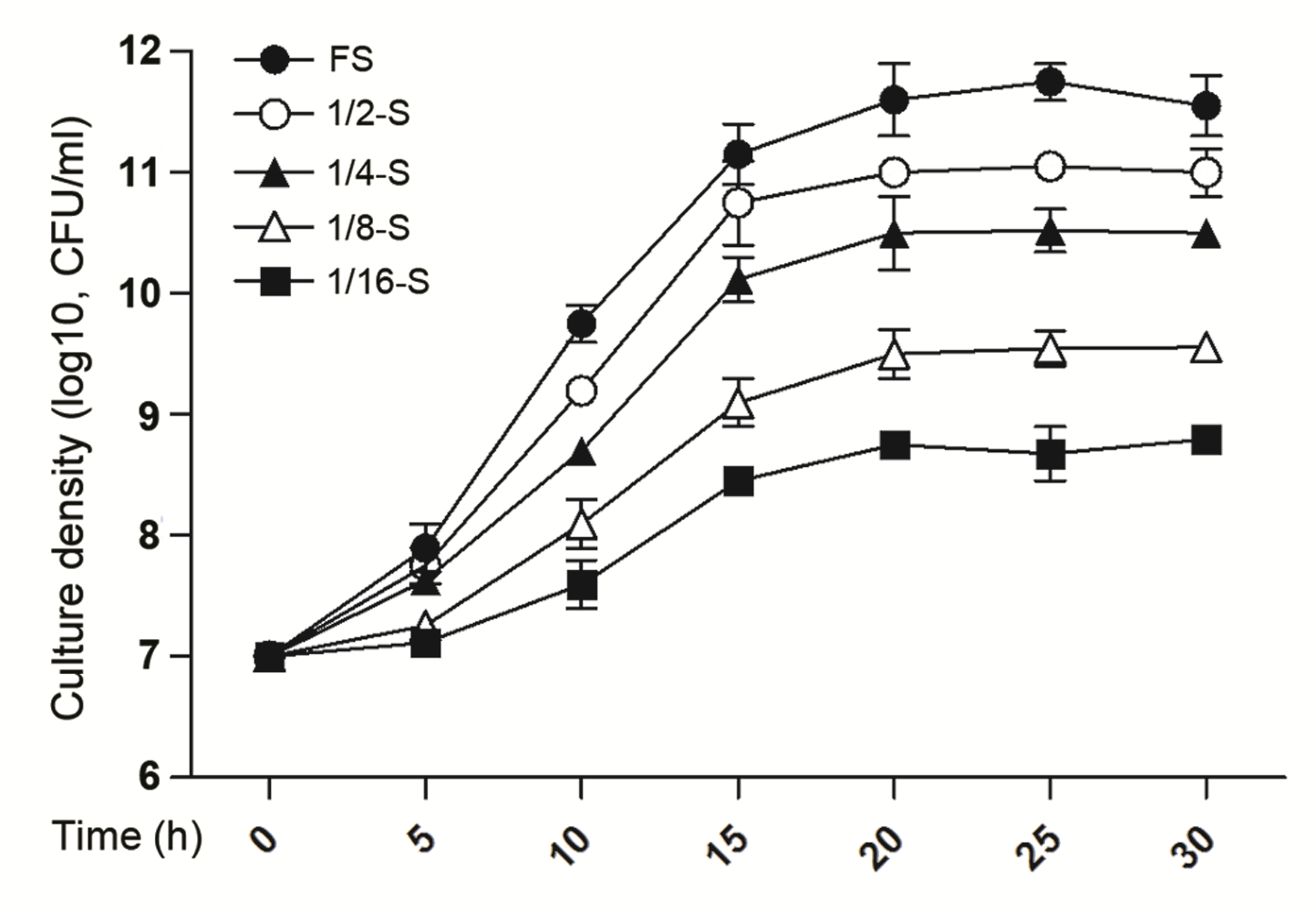


**Supplementary Figure 1.** Growth curves of WT PAO1 in different concentrations of LB medium. Equal amount (1.0 × 10^7^ CFU/ml) of WT PAO1 was inoculated in designated LB medium for 30 h. FS, full-strength LB medium; 1/2-S, 1/2-strength LB medium; 1/4-S, 1/4-strength LB medium; 1/8-S, 1/8-strength LB medium; 1/16-S, 1/16-strength LB medium.


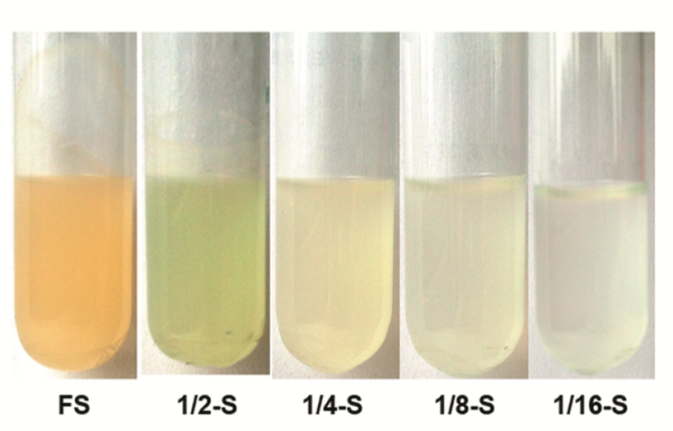


**Supplementary Figure 2.** Liquid color of WT PAO1 overnight cultured in gradient dilutions of LB broth.

**
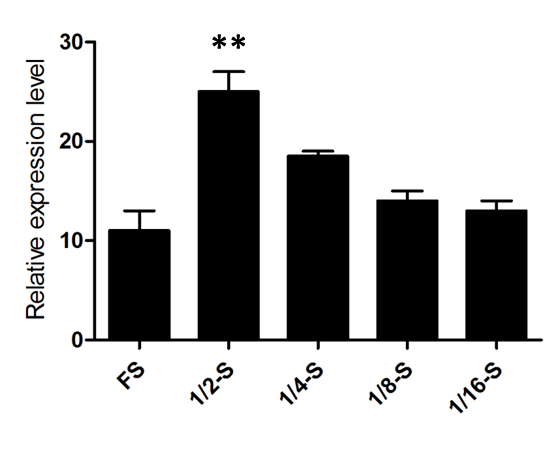
**

**Supplementary Figure 3.** Expression levels of *lasB* when WT PAO1 was cultured in different dilutions of LB broth. Equal amount of WT PAO1 was inoculated in designated LB medium for 16 h. Expression of *lasB* was determined using qPCR. Data are presented as the mean values ± SEM and are representative of three independent experiments. ** *P* < 0.01, One-way ANOVA (Tukey-Kramer post hoc analysis).


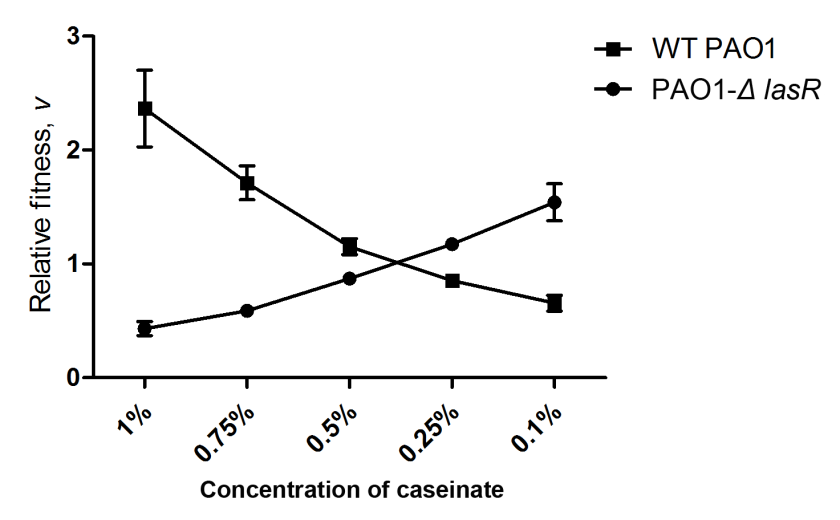


**Supplementary Figure 4.** Relative fitness of WT PAO1 and *lasR* mutant under decreased nutrient supplies. Mixture of *lasR* mutant and WT PAO1 (1:1) was co-cultured in gradient M9-caseinate medium for 36 h. Dash-line indicates the value of *v* = 1. Mean values ± SEM of one experiment are shown and are representative of three independent experiments.


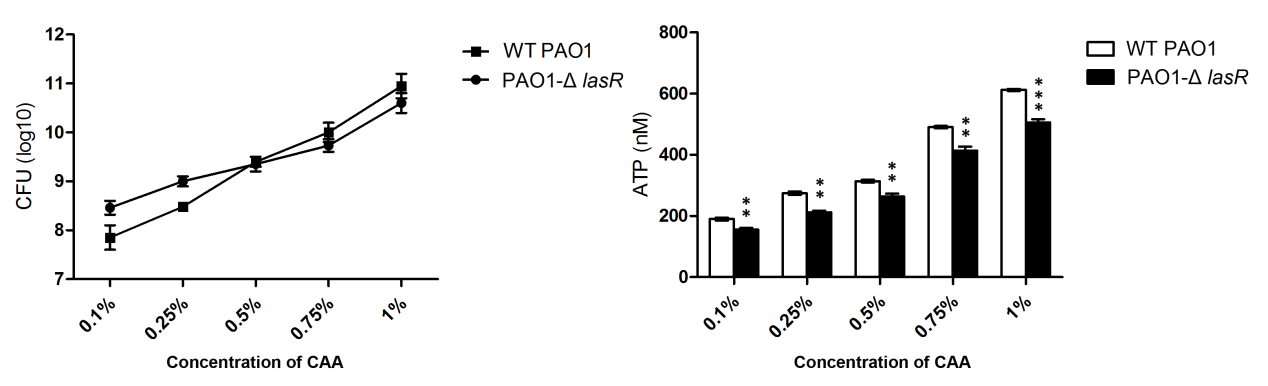


**Supplementary Figure 5.** LasR mutants consume less energy than WT PAO1. (**a**) WT PAO1 and *lasR* mutant were cultured in gradient M9-CAA medium for 24 h. The cell density of each culture was determined by counting the CFUs. (**b**) Production of total ATP normalized with cell density. ATP production was measured by bioluminescence with the ATP Determination Kit (Molecular Probes). Data are presented as the mean values ± SEM and are representative of three independent experiments. **, *P* < 0.01; ***, *P* < 0.001, *t*-test.

## Supplementary Tables

**Supplementary Table 1. Characterization of *lasR* mutants after 30 days long-term normal culture in QS medium.**

| Variants | Day | Mutation^*^ | Change^†^ | Adenosine^‡^ |
| --- | --- | --- | --- | --- |
| *lasR1* | 8 | T→C (+155) | Ile → Thr | – |
| *lasR2* | 12 | Δ (–23 to +9) | Truncation | – |
| *lasR3* | 16 | C→T (+675) | Val→ Ile | – |
| *lasR4* | 20 | G→A (+541) | Glu → Lys | – |
| *ΔlasR* | N/A | Deletion | N/A | – |
| WT PAO1 | N/A | None | N/A | + |

N/A, not applicable.

* Sites of nucleotide substitution relative to translational start site of the *P. aeruginosa* PAO1 *lasR* gene.

† Amino acid changes relative to the LasR protein sequence of *P. aeruginosa* PAO1.

‡ +, positive (wild type phenotype); –, negative (defined *lasR* mutant phenotype).

**Supplementary Table 2.** Characterization of *lasR* mutants after 35 days long-term normal culture in LB broth with shaking.

| Variants | Day | Mutation^*^ | Change^†^ | Adenosine^‡^ |
| --- | --- | --- | --- | --- |
| *lasR5* | 14 | G→A (+203) | Gly → Asp | – |
| *lasR6* | 28 | C→T (+675) | Val→ Ile | – |
| *ΔlasR* | N/A | Deletion | N/A | – |
| WT PAO1 | N/A | None | N/A | + |

N/A, not applicable.

* Sites of nucleotide substitution relative to translational start site of the *P. aeruginosa* PAO1 *lasR* gene.

† Amino acid changes relative to the LasR protein sequence of *P. aeruginosa* PAO1.

‡ +, positive (wild type phenotype); –, negative (defined *lasR* mutant phenotype).

**Supplementary Table 3.** Characterization of biofilm-related *lasR* mutants after 35 days long-term static culture in LB broth.

| Variants | Day | Mutation* | Change^†^ | Adenosine^‡^ |
| --- | --- | --- | --- | --- |
| *lasR7* | 14 | Δ (–23 to +9) | Truncation | – |
| *lasR8* | 28 | G→A (+541) | Glu → Lys | – |
| *lasR9* | 28 | C→T (+675) | Val→ Ile | – |
| *ΔlasR* | N/A | Deletion | None | – |
| WT PAO1 | N/A | None | None | + |

N/A, not applicable.

* Nucleotide substitution or deletion (Δ) at the indicated position relative to translational start site of the *P. aeruginosa* PAO1 *lasR* gene.

† Amino acid changes relative to the LasR protein sequence of *P. aeruginosa* PAO1.

‡ +, positive (wild type phenotype); –, negative (defined *lasR* mutant phenotype).

**Supplementary Table 4. Partial genes with differential expression when WT PAO1 was cultured in gradient dilutions of LB broth. Data are presented as median-centered log_2_ (FPKM) (*P* < 0.05). DNA mismatch repair genes and partial QS-inducible genes were highlighted in bold.**

| Gene | Full-strength LB | 1/2-strength LB | 1/4-strength LB | 1/8-strength LB |
| --- | --- | --- | --- | --- |
| ***dinB*** | 0.936996315 | 1.874248311 | -0.289717195 | -2.521527432 |
| ***mutL*** | 2.055106042 | 0.587473289 | 1.044671225 | -3.687250557 |
| ***mutY*** | 1.791937616 | -0.597312539 | -0.597312539 | -0.597312539 |
| ***mutS*** | 3.106411753 | -1.035470584 | -1.035470584 | -1.035470584 |
| ***mutM*** | 3.251564205 | -1.083854735 | -1.083854735 | -1.083854735 |
| ***lasA*** | 0.903282544 | 2.550438096 | -0.282591944 | -3.171128696 |
| ***lasB*** | -5.604161858 | 3.510155709 | 1.115487069 | 0.978519081 |
| ***rhlA*** | -1.487768289 | 0.466332821 | 1.132504382 | -0.111068914 |
| ***rhlB*** | -2.955054215 | 1.685821175 | 0.879606443 | 0.389626597 |
| ***aprA*** | -1.478651073 | 2.343678073 | -1.48560217 | 0.62057517 |
| ***hcpB*** | 1.085083291 | 1.610350545 | -3.519278678 | 0.823844842 |
| ***hcpC*** | 0.023764462 | 0.620227438 | -1.427870777 | 0.783878877 |
| ***napA*** | 0.298951278 | 1.135182404 | 0.619862969 | -2.053996651 |
| ***coxA*** | -2.231324931 | 0.778693526 | 1.013641707 | 0.438989697 |
| ***coxB*** | -2.061303808 | 0.46448117 | 1.139445115 | 0.457377523 |
| ***tadB*** | -3.409808586 | 1.23620292 | 1.5481763 | 0.625429365 |
| ***tadZ*** | -3.194147737 | 2.769885785 | 0.616285083 | -0.192023131 |
| ***tadC*** | -4.629356639 | 2.194354514 | 2.093969828 | 0.341032297 |
| ***tadD*** | -4.711014223 | 2.698232436 | 1.691254312 | 0.321527474 |
| ***tadA*** | -1.547879806 | 4.643639418 | -1.547879806 | -1.547879806 |
| ***tadG*** | -1.300847279 | 3.902541838 | -1.300847279 | -1.300847279 |
| ***exbD1*** | -1.975398367 | 3.198324735 | -1.975398367 | 0.752472 |
| ***exbB1*** | -0.781308625 | 2.343925874 | -0.781308625 | -0.781308625 |
| ***phzE2*** | -3.210052668 | 1.318047284 | 1.051300989 | 0.840704395 |
| ***phzD2*** | -3.10959362 | 1.411645014 | 0.893132091 | 0.804816515 |
| ***phzG2*** | -2.275332093 | 1.477123062 | 0.520491135 | 0.277717896 |
| ***phzC1*** | -5.375990183 | 1.926986815 | 2.14004861 | 1.308954757 |
| ***phzF1*** | -5.163298284 | 1.738980346 | 1.790188139 | 1.634129798 |
| ***phzD1*** | -5.201618675 | 1.792293399 | 1.845581597 | 1.56374368 |
| ***phzE1*** | -4.772377945 | 1.292595417 | 2.006894909 | 1.472887619 |
| ***phzG1*** | -4.631940171 | 2.086996774 | 1.602957713 | 0.941985683 |
| ***phzF2*** | -3.882374744 | 1.640558733 | 1.140143998 | 1.101672013 |
| ***pqqC*** | -1.962307356 | 2.429639041 | 1.494975671 | -1.962307356 |
| ***pchB*** | -1.653972504 | 4.961917512 | -1.653972504 | -1.653972504 |
| ***pchA*** | -1.518974598 | 4.556923795 | -1.518974598 | -1.518974598 |
| ***pfpI*** | 1.191017961 | 3.232120363 | -2.211569162 | -2.211569162 |
| ***phnA*** | 0.590263332 | 2.897564158 | -3.920084679 | 0.432257189 |
| ***pqsA*** | 0.557071639 | 1.846177431 | -0.838961426 | -1.564287643 |
| ***arcA*** | 0.269758886 | 1.800684207 | -0.16585436 | -1.904588733 |
| ***arcC*** | 0.019702193 | 1.824230284 | -0.485108769 | -1.358823707 |
| ***arcB*** | 0.125459366 | 1.741184753 | -0.353492304 | -1.513151816 |
| *grpE* | 0.396148895 | 1.843617372 | -1.517547648 | -0.722218618 |
| *PA2197* | 0.241718897 | 2.018943014 | -1.549570335 | -0.711091576 |
| *PA1299* | -0.282012905 | 2.990926547 | -2.00363055 | -0.705283092 |
| *dnaK* | 0.178808784 | 2.426164061 | -1.82285501 | -0.782117836 |
| *htpG* | 1.26069029 | 3.45722101 | -4.049867964 | -0.668043336 |
| *PA0449* | 1.064233506 | 3.006958428 | -3.773806263 | -0.297385671 |
| *PA0779* | 0.699266835 | 3.743878624 | -4.622863223 | 0.179717763 |
| *hslV* | 0.508895244 | 3.96631987 | -3.339732703 | -1.135482411 |
| *hslU* | 0.576712594 | 3.912809363 | -3.792048772 | -0.697473185 |
| *dnaJ* | 0.304065283 | 1.706716829 | -1.233993886 | -0.776788226 |

**Supplementary Table 5.** Mutation frequencies of *lasR* mutant isolates after exposure to rifampin.

| Variant | Mutation^*^ | Change^†^ | Mutation frequencies |
| --- | --- | --- | --- |
| *lasR1* | T→C (+155) | Ile → Thr | 7.3 × 10^-5^ |
| *lasR2* | Δ (–23 to +9) | Truncation | 6.3 × 10^-5^ |
| *lasR3* | C→T (+675) | Val→ Ile | 4.9 × 10^-5^ |
| *lasR4* | G→A (+541) | Glu → Lys | 5.4× 10^-5^ |
| *lasR5* | G→A (+203) | Gly → Asp | 2.7 × 10^-5^ |
| *ΔlasR* | N/A | Deletion | 0.9 × 10^-5^ |
| WT PAO1 | None | None | 1.67 × 10^-5^ |

N/A, not applicable.

* Sites of nucleotide substitution relative to translational start site of the *P. aeruginosa* PAO1 *lasR* gene.

† Amino acid changes relative to the LasR protein sequence of *P. aeruginosa* PAO1.

**Supplementary Table 6. Primers used in this study.**

| Gene assayed | Primer sequence |
| --- | --- |
| *lasR-full* | Forward: 5’- CGCCGAACTGGAAAAGTGGC -3’ |
|  | Reverse: 5’- TGAGAGGCAAGATCAGAGAG -3’ |
| *lasR-RT* | Forward: 5’- CTTCATCGTCGGCAACTAC -3’ |
|  | Reverse: 5’- GTCTGGTAGATGGACGGTTC -3’ |
| *lasB* | Forward: 5’- ATCGGCTACGACATCAAGAAGG -3’ |
|  | Reverse: 5’- CCGCTGTTGTAGTTGCTGGTG -3’ |
| *rpoS* | Forward: 5’- GCCTGAACGAACGGGTGACT -3’ |
|  | Reverse: 5’- CACCTCACGCTGCTTGTCG -3’ |
| *dinB* | Forward: 5’- GGCTTGCCTGGAGGAACTG -3’ |
|  | Reverse: 5’- GGTCTGGGTGAAATCGTGAA-3’ |
| *mutS* | Forward: 5’- GTAAATCCACCTACATGCGGC -3’ |
|  | Reverse: 5’- CTCTTGTCGGTGGCGTTGTG -3’ |
| 16S rRNA | Forward: 5’- GGACGGGTGAGTAATGCCTA -3’ |
|  | Reverse: 5’- CGTAGGAGTCTGGACCGTGT -3’ |
